# Psychometric properties of the Sinhala version of the PedsQL™ 4.0 Generic Core Scales in early adolescents in Sri Lanka

**DOI:** 10.1186/1477-7525-10-105

**Published:** 2012-09-04

**Authors:** Manjula Nishanthi Danansuriya, Lalini C Rajapaksa

**Affiliations:** 1 School & Adolescent Health Unit, Family Health Bureau, Ministry of Health, 231, De Saram Place, Colombo 10, Sri Lanka; 2 Department of Community Medicine, Faculty of Medicine, University of Colombo, Colombo, Sri Lanka

**Keywords:** PedsQL™, Sinhala, Sri Lanka, Adolescents, Children, Health Related Quality of Life, Psychometrics

## Abstract

**Background:**

The concept Health related Quality of life (HRQOL) is increasingly recognized as an important health outcome measure in clinical and research fields. The present study attempted to evaluate the psychometric properties of the Sinhala version of the Pediatric Quality of Life Inventory™ 4.0 (PedsQL™ 4.0) Generic Core Scales among adolescents in Sri Lanka.

**Methods:**

The original US PedsQL™ was translated into Sinhala and conceptually validated according to international guidelines. A cross-sectional study was conducted among 142 healthy school going adolescents (12-14 years), their parents (n = 120) and a group of adolescents with asthma who attended asthma clinics (n = 115). Reliability was assessed using Cronbach’s alpha and validity by examining scale structure, exploring inter-scale correlations and comparing across known groups (healthy vs. chronically ill).

**Results:**

The PedsQL™ Sinhala version was found to be acceptable with minimal missing responses. All scales demonstrated satisfactory reliability. Cronbach’s alpha for the total scale scores was 0.85 for adolescent self-report while for the parent proxy-report for the healthy group it was 0.86. No floor effects were observed. Ceiling effects were noticed in self-report and parent proxy-report for the healthy group. Overall results of the multi trait scaling analysis confirmed the scale structure with 74% item-convergent validity, 88% item-discriminant validity and an overall scaling success of 72%. Moderate to high correlations were shown among the domains of teen self-report (Spearman rho = .37-.54) and between teen self-report and parent proxy-reports (Spearman rho = .41-.57). The PedsQL™ tool was able to discriminate between the quality of life in healthy adolescents and adolescents with asthma.

**Conclusion:**

The findings support the reliability and validity of the Sinhala version of the PedsQL™ 4.0 Generic Core Scales as a generic instrument to measure HRQOL among early adolescents in Sri Lanka in a population setting.

## Background

Advances in biomedical science and technology have resulted in increased survival among pediatric patients with acute and chronic diseases in most countries. With this improvement in life status, health-related quality of life (HRQOL) issues have become more important, particularly since this survival is accompanied by significant ongoing healthcare needs. Patient’s perspectives and their values measured in various forms such as quality of life or disability adjusted life years are being recognized as an important in health care decisions since they account for both objective and subjective experiences of the disease [1-4].

Although HRQOL has been explored previously among adults in Sri Lanka with various disease conditions like cancer, cataract and filarial lymphoedema; research among adolescents is limited [5-8]. The period of adolescence is generally considered as a healthy period of life. However, due to the transitional nature of the adolescent period, they are more likely to experience a range of physical and psychosocial health issues, warranting the need to assess their HRQOL.

Various generic and disease-specific HRQOL instruments have been developed specifically for children and adolescents [9]. The Paediatric Quality of Life Inventory™ (PedsQL™) was selected for this study considering its brevity, ease of administration, availability of age appropriate versions and the strong psychometric properties reported internationally. The PedsQL™ 4.0 Generic Core Scales has been validated in different settings with different populations [10-15] Furthermore, the PedsQL™ 4.0 includes all of the core dimensions of health delineated by World Health Organization, including school (role) functioning [16].

The objective of the present study was to assess the reliability and validity of Sinhala version of PedsQL™ 4.0 Generic Core Scales in a sample of 12-14 year old healthy school children and children with a known chronic disease condition (asthma).

## Methods

### Measures

#### PedsQL ™ 4.0 Generic Core Scales

The PedsQL™ 4.0 Generic Core Scales consists of 23 items grouped in to four domains: Physical Functioning (PF), Emotional Functioning (EF), Social Functioning (SF) and School Functioning (SchF). It has parallel child self-report and parent proxy-report formats for separate age groups [17,18]. We have selected the adolescent version (13–18 years) for the present study. It assessed how much of a problem each item has been during the past month and responses are made on a 5 point scale ranging from 0 (never a problem) to 4 (almost always a problem).

Items are reverse scored and linearly transformed to a 0–100 scale and higher scores indicate better HRQOL. According to the instructions given with the PedsQL™, scale scores are computed as the sum of the items divided by the number of items answered (accounting for missing data). If more than 50% of the items in the subscale are missing, the scale score is not computed. The Physical Health Summary Score (8 items) is the same as the Physical Functioning Scale. The Psychosocial Health Summary Score is computed as the sum of the items divided by the number of items answered in the Emotional, Social and School Functioning Scales [17].

The PedsQL™ 4.0 Generic Core Scales teen and parent versions were translated into Sinhala and linguistically validated according to international guidelines and in close collaboration with the first author [19,20]. Two independent translators (A Consultant Community Physician and a health Education officer), both fluent in Sinhala and English languages, translated the tool to Sinhala. The principal investigator (PI) reviewed the two drafts and formed a combined version. Where there were differences, consensus was achieved by a discussion involving the two translators and the PI. In some cases, the opinions of experts/target group were also sought. Another two translators (A Consultant Community Physician and a University lecturer), both fluent in Sinhala and English languages, independently translated each questionnaire to English. None of them had access to the original English version of PedsQL™ modules. The principal investigator (PI) compared the drafts and formed a combined English version and evaluated it with the original English questionnaire and necessary modifications were carried out in the Sinhala translation accordingly. The provisional Sinhala translation was pre tested using cognitive debriefing interviews among teens and parents following the guidelines. Following the necessary changes, the final Sinhala version of PedsQL™ was formulated.

### Additional survey items

The students completed additional items on basic socio-demographic information; present and past medical history (presence of any chronic diseases/acute severe illnesses during the preceding one month); number of days and reasons for school absence during the previous month, and the number of visits made to any health facility or General Practitioner (GP) due to illness during the preceding month.

### Participants and settings

The present school based study was carried out as a component of a research study which explored the prevalence, correlates of asthma among 12-14 year school children and HRQOL among students identified with asthma. Reliability & validity of PedsQL™ 3.0 Asthma module has been published elsewhere [21]. The results of the pilot study, showed satisfactory level of acceptability and level of comprehensiveness for the Sinhala version of PedsQL™ Generic Core Scale teen version among 12 year olds. It was difficult to include the students from higher grades due to examinations and rigid class timetables. Therefore it was decided to utilize teen version (13–18 years) to examine the HRQOL among 12–14 year olds in the present study with the approval of the first author of the tool, Prof.J.W. Varni.

The Sinhala version of PedsQL™ 4.0 Generic Core Scales was tested as self administered tools to school children aged 12–14 years and their parents. Sample size was calculated as 115 based on the testing of hypothesized scale structure of the questionnaires using multivariate analysis technique [22]. The school children were selected from four classes in two Sinhala medium schools both randomly selected from one geographical area in the district of Gampaha. Two trained research investigators (RI) (newly qualified medical graduates) visited each class room on a prior day, described the purpose of the study and distributed parent consent forms and PedsQL*™* parent proxy-reports, to be completed by parents. The class teacher was asked to collect the consent forms and completed parent forms. On the day of the study, two research investigators and the principal investigator (MND) visited the selected class, checked for parental written consent forms and distributed the questionnaires after obtaining the verbal consent from the students. All the students were invited to participate. They completed confidential, coded, self administered questionnaire in the classroom. Exclusion criteria included: physical or mental disability; suffering from any chronic or acute severe illness during the previous one month; lack of student’s or parental consent.

In the assessment of known-groups discriminant validity, adolescents aged between 12–14 years, physician-diagnosed cases of asthma were recruited. Adolescents with asthma and their parents were accrued from asthma clinics in a consecutive manner from four university hospitals selected on convenience basis. Physically or mentally disabled patients; adolescents suffering from any other chronic disease in addition to asthma; adolescents/parents who could not understand the Sinhala language and those adolescents or their parents declining participation were excluded from the study. Adolescents and their parents were given the PesdQL*™* Sinhala version during the waiting time in clinics, before the consultations and asked to complete them independently. The investigators were present throughout the process.

### Psychometric testing of the validity of PedsQL*™* 4.0 Generic Core Scales-Sinhala version

The feasibility of the PedsQL™ 4.0 Generic Core Scales was assessed using the percentage of missing values and rate of completion of scales [11,12,15]. Range of measurement was assessed based on ceiling effect and floor effects.

Conceptual validity was established by the assessment of hypothesized scale structure using multi trait scaling analysis and by assessment of construct validity. Multi-trait scaling analysis is used to examine the extent to which the items of questionnaire could be combined in to the hypothesized multi – item scales based on the evaluation of item-scale correlations [17,23]. Item convergence was defined as a correlation of 0.40 or greater between an item and its own scale (corrected for overlap). Item discrimination was based on a comparison of the magnitude of the correlation of an item with its own scale compared with other scales. Scaling successes were defined as those cases in which an item correlated significantly higher (more than 1.96 standard errors) with its own scale (corrected for overlap) than with another scale [17,23].

Construct validity was evaluated by assessment of convergent and divergent validity using inter-scale correlations and comparing the known groups. During the assessment of inter scale correlations, we hypothesized that conceptually related scales (e.g. PF and SF scales and SF and SchF scales) would correlate significantly with one another and those scales with less in common would have lower correlations according to previous literature [12,13]. Medium to large sized (>0.30) correlations were expected in total scores and similar domain scores (mono trait-hetero method) between PedsQL self and parent reports. It is also expected a higher correlations in SF and SchF domains and lower correlations among EF domains in keeping with previous PedsQL studies [11,12].

The known group method compared the scale scores between clinically different “Healthy adolescents” and “adolescents with a chronic disease (asthma)”. We anticipated that healthy adolescents would report higher scores compared to adolescents with asthma based on previous literature [17,24].

Reliability of the PedsQL™ Generic Core Scale was examined through internal consistency measures. Test-retest reliability was not assessed, as HRQOL is a changing phenomenon.

### Statistical analysis

Scale internal consistency reliability was determined by calculating Cronbach’s alpha [25]. The PedsQL scores between healthy and asthmatics were compared using Mann Whitney *U* test. Concordance between scores was determined using Spearman rho. Correlations are designated as small (0.10-0.29), medium (0.30-0.49) and large (> = .50) as suggested by Cohen in 1988 [18]. During interpretation of the Spearman rho correlations, identical values with Pearson r were considered as equivalent values. Data were analysed using SPSS version 15.0(SPSS Inc., Chicago, IL, USA). For all analysis, p < 0.05 was considered statistically significant.

## Ethics

No major ethical issues were identified in this study design. The ethical approval was obtained by the Ethics Review Committee of Faculty of Medicine, University of Colombo, Sri Lanka.

## Results

### Sample characteristics

Of a total of 190 students approached, 176 were present on the day of the study. Three students lacked parental consent and were excluded. None of the students refused participation in the study. Data collection was carried out from February to April, 2008. The mean age was 12.8 years (SD = 0.8). Majority of the sample were Sinhalese (91.6%), living with both parents (83.2%) and had parent reports available (83.8%). Categorization of students as “healthy” and “not healthy” was done according to the student’s self report. Of the 173, fourteen had chronic disease conditions like asthma, chronic headache, epilepsy etc. Another 15 reported to have acute disease conditions such as dengue, chicken pox, sports/accidental injury and/or one or more visits to General Practitioner during the preceding one month. There were two students (1.1%) with reading and/or writing difficulty to whom the questionnaire was interviewer administered by a research assistant. Finally, all 31 (17.9%) above, were excluded in the analysis leaving 142 “healthy” students.

Of these 142 respondents identified as healthy, parent reports were available only in 120 (84.5%). Thus 142 healthy students and 120 parents are included in the analysis. There were 74 boys (52%). There were no significant differences observed between the selected sample (n = 142) and the original respondents (n = 173) with regard to sex, race, living arrangements and availability of parent reports. The mean age for “asthma” group was 13.0(SD = 0.9) years. The characteristics of “Healthy” and “Asthmatic” groups were examined to elicit their comparability, as they were drawn from two different settings. There were no significant differences observed with regard to sex, mean age, grade, ethnicity, family monthly income level or parents’ education level (Table 1).

**Table 1:** Socio-demographic characteristics of healthy and asthma groups

	**Healthy sample (n = 142)**		**Asthma sample (n = 115)**		**Significance**
	**N**	**(%)**	**N**	**(%)**	
Sex					
Male	74	52.1	65	56.5	*X*^2^ = .33
Female	68	47.9	50	43.5	p = .5
Grade					
7	47	33.1	34	29.6	*X*^2^ =3.1
8	53	37.3	35	30.4	p = .2
9	42	29.6	46	40.0	
Race					
Sinhala	130	91.6	93	86.1	*X*^2^ =6.8
Tamil	4	3.4	4	3.5	p = .07
Muslim	7	4.2	13	7.0	
other	1	0.7	4	3.5	
Mean Age	12.8 yrs		13.0 yrs		p = .09
Parent Education Level					
No schooling	5	4.3	2	2.3	
Primary	10	8.5	6	7.0	*X*^2^ =2.0
Secondary	45	38.5	40	46.5	p = .8
Passed O/L	31	26.5	23	26.7	
Passed A/L	15	12.8	9	10.5	
Higher Education	11	9.4	6	7.0	
Missing	25	17.6	29	25.2	

*Significance level: p < 0.05 (two tailed test).

### Feasibility

The percentage of missing responses for student-self report at item level was 0.39%. The time taken to complete the teen-report ranged from 5 to 8 min. There was a 100% response rate for all domains of self report with no missing data. It was not possible to assess the time taken to complete the parents’ reports as they were sent home for completion. However the percentage of missing data was high compared to teen-self reports which ranged from 6.6% to 7.5% at domain level and 7.6% at item level.

### Descriptive statistics

The PedsQL™ Generic total scores were observed to have a negatively skewed distribution for both teen and parent formats. The full range of item responses in the scale had been used in most of the domains. Social Functioning (SF) scale in self report and physical functioning (PF) in parent report had the highest mean scores while emotional functioning (EF) had the lowest scores in both reports.

If more than 15% of respondents used extreme values, it was considered as presence of ceiling and floor effects [26]. SF domain in the self report and PF,SF and SchF domains in parent reports showed ceiling effects while none showed floor effects (Table 2).

**Table 2:** **Scale Descriptive statistics for PedsQL**™ Generic Core Scales –self report and parent proxy-report (healthy & asthma groups)*

**Scale**	**N**	**Missing N (%)**	**Mean (SD)**	**Median**	**Range**	**% Floor/% Ceiling**	**Reliability (Cronbach’s alpha)**
**Self Report**							
*Total score*	257	0(0.0)	87.2(10.4)	83.7	54.3-100.0	0.0/1.9	.85
Physical functioning	257	0(0.0)	84.8(12.3)	87.5	28.1-100.0	0.0/10.5	.74
*Psycho social health*	257	0(0.0)	81.5(11.9)	83.3	51.7-100.0	0.0/2.7	.82
Emot. Functioning	257	0(0.0)	78.9(14.3)	80.0	35.0-100.0	0.0/8.9	.60
Social Functioning	257	0(0.0)	86.0(13.6)	90.0	30.0-100.0	0.0/24.5	.65
School Functioning	257	0(0.0)	79.5(16.6)	85.0	25.0-100.0	0.0/13.2	.77
**Parent Report**							
*Total score*	117	25(17.6)	87.9(11.9)	90.2	30.0-100.0	0.0/7.0	0.86
Physical functioning	112	30(21.2)	91.6(9.6)	93.7	59.3-100.0	0.0/21.5	0.77
*Psycho social health*	115	27(19.5)	86.0(13.6)	88.3	12.5-100.0	0.0/7.0	0.82
Emot. Functioning	112	30(21.2)	84.4(15.1)	90.0	30.0-100.0	0.0/14.8	0.68
Social Functioning	111	31(21.8)	90.2(13.2)	95.0	35.0-100.0	0.0/33.0	0.67
School Functioning	111	31(21.8)	86.7(13.9)	90.0	30.0-100.0	0.0/17.6	0.77

*Analysis based on 257 self reports (healthy & asthma groups) and 120 parent reports of healthy adolescents.

*PF* Physical Functioning, *EF* Emotional Functioning,*SF* Social Functioning, *SchF* School Functioning.

### Validity and reliability of PedsQL™ Generic Core Scale

Content validity was confirmed by an expert panel consisted of paediatricians, community physicians, school teachers, parents and school going adolescents. Each item was evaluated for its relevance, appropriateness of the wording used and acceptability in the local context. Ratings were made using 10 point scale (0- worst, 10 -best). Individual scale reliabilities have exceeded 0.6, the minimum criterion considered for exploratory purposes. Reliability values for PedsQL™ total score for both self report and for parent report, have exceeded the recommended minimum alpha coefficient, 0.70 for group comparisons [27] confirming the reliability of PedsQL™ Generic Core Scale - Sinhala version.

Item-convergent validity was confirmed for the majority of items in three of the four hypothesized domains (PF, SF and SchF). Four items in the EF domain, (QOL9- feeling afraid; QOL11- feel angry; QOL12- trouble sleeping; QOL13- worried about what will happen) and one item in the SF domain (QOL14: trouble getting along with other teens) failed to show item convergent validity (Table 3).

**Table 3:** **Item-Scale correlations for multitrait-scaling analysis of PedsQL**™ **Generic Core Scales – self-report for healthy & asthma groups (corrected for overlap)**

**Item NO.**	**PF**	**EF**	**SF**	**SchF**
PF				
QOL1	**.42**	.21	.30_$_	.26
QOL2	**.44**	.18	.33_$_	.28
QOL3	**.53**	.14	.26	.29
QOL4	**.39**	.13	.31_$_	.22
QOL5	.33	.13	.21_$_	.07
QOL6	**.48**	.21	.26	.21
QOL7	**.45**	.25	.19	.21
QOL8	**.43**	.32_$_	.23	.19
EF				
QOL9	.16	.37	.17	.25_$_
QOL10	.25	**.51**	.25	.19
QOL11	.16	.37	.25_$_	.16
QOL12	.25_$_	.27	.39***_$_	.29***_$_
QOL13	.22***_$_	.20	.34***_$_	.32***_$_
SF				
QOL14	.33***_$_	.32***_$_	.27	.29***_$_
QOL15	.31_$_	.28	**.45**	.23
QOL16	.25	.27	**.46**	.30
QOL17	.23	.31	.**47**	.47_$_
QOL18	.22	.22	**.39**	.37_$_
SchF				
QOL19	.27	.33	.36	**.52**
QOL20	.26	.41	.39	**.55**
QOL21	.24	.32	.38	**.53**
QOL22	.30	.14	.33	**.56**
QOL23	.30	.19	.36	**.57**

N=257.

Item- scale correlations with item- convergent validity are shown in bold typing.

Failed tests with item convergent validity are shown as underlined.

*** - failed tests with item discriminant validity, where an item correlated higher with a scale other than its own.

N$- Scaling errors, where an item correlated with a scale other than its own, with a standard error of correlation more than 1.96 (r>1.96 SE).

Of the 69 tests of item correlation with a scale other than its own, 61 tests (88%) demonstrated item- discriminant validity. Scaling success was noted in 50 of 69 tests (72%). Several items (QOL 12, 13 and 14) showed low discriminant validity. In summary, 17 of 23 items (74%) exhibited item-convergent validity and 88% of items showed item-discriminant validity with 72% scaling success, supporting the evidence that the PedsQL™ Generic Core Scales- Sinhala version has satisfactory convergent and discriminant validity.

The assessment of correlations between the domains of teen self report revealed moderate to large correlations between PF and SF (ρ = .44) domains (Table 4). Moderate teen-parent correlation was demonstrated between total scale scores (ρ = .45). Our results show that monotrait-hetero method correlations were higher than multitrait-hetero method correlations confirming the convergent and divergent validity of the tool [28]. Higher inter-correlations were seen in SF and SchF domains while lower correlations in EF domain in keeping with previous PedsQL™ studies [11,12].

**Table 4:** Inter-correlations for the PedsQL™ 4.0 Generic Core Scales adolescent self-report and parent proxy-report (Spearman rho) for healthy & asthma groups

	**Self report**				**Parent report**		
**Self report**	**PF**	**EF**	**SF**	**SchF**	**PF**	**EF**	**SF**
PF							
EF	.37^$^						
SF	.44^$^	.46^$^					
SchF	.42^$^	.43^$^	.54^$^				
Parent report							
PF	.44	.22	.40	.23			
EF	.44	.41	.33	.18*	.53		
SF	.34	.24	.57	.26	.43	.57	
SchF	.30	.35	.40	.56	.43	.47	.44

$ : N =257 (142 healthy & 115 asthma) all other N=115 (Parent reports of healthy teens; missing =5).

All correlations are significant at the 0.05 level (2-tailed) except*

Mono trait – hetero method correlations are in Bold.

*PF* Physical Functioning, *EF* Emotional Functioning, *SF* Social Functioning, *SchF* School Functioning.

Group comparison shows that the PedsQL™ total and domain scores were significantly higher among healthy students compared to those with asthma. The results confirm the ability of PedsQL™ Sinhala version to differentiate clinically distinct groups (Table 5). It shows significantly lower scores in all domain scores and total scores among adolescents with asthma compared to their healthy peers.

**Table 5:** **Mann Whitney *U *test values comparing healthy and asthma groups –teen report**

	**Healthy sample (N = 142)**	**Asthma sample (N = 115)**	**P value**
**Scale**	**Mean (SD)**	**Mean (SD)**	
*Total score*	87.2(9.3)	76.3(9.0)	0.001
PF	88.7(11.0)	78.8(11.1)	0.001
*Psycho social health*	86.3(10.2)	74.9(11.2)	0.001
EF	81.1(14.9)	74.3(14.1)	0.006
SF	89.9(11.5)	81.3(12.0)	0.001
SchF	87.8(11.2)	69.1(17.1)	0.001

*PF* Physical Functioning; *EF* Emotional Functioning; *SF* Social Functioning; *SchF* School Functioning.

## Discussion

This study provides initial evidence regarding the validity and reliability of the PedsQL™ 4.0 Generic Core Scale –Sinhala version as a measure in evaluating quality of life among adolescents in a population setting.

The overall picture shows that the PedsQL™ Generic Core Scale –Sinhala version has better acceptability among adolescents with minimum missing values at item and scale level. Similar results have been reported with studies carried out in Norway [12], Argentina [15], Brazil [29], and Austria [30].

However, low levels of missing values and high response rate in self reports may have been due to the fact that research investigators present on site checked for missing answers at the end and returned it to the relevant students for completion. Higher missing values for parent proxy-reports may have been due to differences in mode of administration and variability in parental literacy or education levels.

The frequency distribution of individual items revealed that the full range of 0–100 had been used in a majority of the items in the self report. Comparable results have been presented in UK [11] and USA [18]. The distribution of responses showed an asymmetrical and negatively skewed pattern, as expected with a healthy population [17].

The present study showed that SF domain in the self and parent-proxy report had ceiling effects, replicating the results of the studies done in UK [11], Greece [13], Korea [14], and original PedsQL™ validation study [17]. The ceiling effects can be expected with generic HRQOL instruments since they are designed to apply to a wide range of population groups. Absence of floor effects in this group could have been due to inclusion of relatively healthy school going adolescents along with the teens from asthma clinics that are likely to have well controlled disease condition, compared to inward patients.

The mean total score was 87.2 and this is in accordance with studies done among school children in Korea [14] and Brazil [29]. However, most other PedsQL™ studies reported relatively lower mean scores [11,13,15,31]. The reasons for a higher mean score in the present study could be due to cultural differences in perception of issues related to HRQOL.

In the assessment of the hypothesized scale structure item-convergent validity was confirmed for majority of items in the five hypothesized scales. Failure in convergence with several domains may be due to the overlapping nature of the construct with other related but distinct domains. For example: “trouble getting alone with other kids” showed higher correlations with other scales. Students could have perceived that as a limitation of physical activities or emotional wellbeing rather than attributes of social functioning which prevented them performing as their peers. All items in the SchF domain showed convergent and discriminant validity. It also highlights the importance of inclusion of the role (school) functioning concept in measuring multi dimensional HRQOL.

The inter scale correlations between the domains of the PedsQL™ self report were consistent with the hypothesis that conceptually related scales (e.g. Social and School domains) would correlate significantly with one another (with Spearman rho/Pearson r ≥ 0.40).

The correlations between teen and parent ratings were higher with more observable SF and SchF domains compared to internal constructs as EF. A similar pattern has been reported in Japan [31], Sweden [32] and with the Dutch translation [33]. The overall inter scale correlations between teen and parent reports (ρ = 0.41-0.56) are within the range reported elsewhere [12,14,15,17]. Low teen parent concordance may be due to the widening communication gap observed in this age group. Adolescence is a period of transition during which they try to explore their identities and independence as individuals [34]. It also confirms the need for a separate child/teen self report in the evaluation of HRQOL among children and adolescent in clinical practice.

Known group comparisons showed that the instrument had the ability to discriminate between healthy and adolescents with asthma adding to the growing body of HRQOL literature, about the discriminatory ability of PedsQL™ [4,14,15,17,24,35].

Reliability coefficients for total scale in both teen and parent reports have exceeded the recommended minimum alpha coefficients of 0.70 for group comparisons verifying the reliability of the PedsQL™ Sinhala version. Cronbach’s alphas for domains were comparable to the findings in Iran [4] Brazil [29] and Argentina [15]. However, they were low compared to other studies [11-13]. This may be due to the comparatively smaller sample size of the present study. Test –retest reliability was not measured as HRQOL scores are expected to change over time limiting its validity as a measure of reliability [34].

The overall results exhibit that the PedsQL™ Generic Core- Sinhala version has a good level of validity and reliability, among Sri Lankan school going adolescents.

The current study has few potential limitations. The generalizability of the study findings is limited as we restricted the study population to 12–14 year old adolescents in Sinhala medium schools due to constraints in resources. Therefore the inferences may not be applicable to adolescents belonging to other ethnic groups or adolescents outside this age limits and for non school going children. The self report questionnaires were administered in normal classroom environment. There may be factors that can influence the results which we have not accounted for. The sample size for healthy and asthma group although calculated on a statistical basis, was small compared to other PedsQL studies. Thus, comparison of results with other studies should be done with caution. Sensitivity of the tool with the changes over time could not be measured due to the cross-sectional nature of the study design.

## Conclusion

The present study provides evidence of the usefulness of the PedsQL™ Generic Core-Sinhala version as a population health measure in school setting in Sri Lanka. Further studies with larger sample, wider age range may facilitate evaluation of applicability of PedsQL™ Generic Core-Sinhala version in clinical settings.

## Abbreviations

HRQOL: Health related quality of life; PedsQL™: Paediatric quality of life inventory™; PF: Physical functioning; EF: Emotional functioning; SF: Social functioning; SchF: School functioning; PI: Principal investigator.

## Competing Interests

Authors have no competing interests.

## Authors’ contributions

MND contributed in designing the study, preparing the proposal, data collection, data analysis and wrote the manuscript. LCR participated in design phase, data analysis, writing the manuscript and critically revised the manuscript. All authors read and approved the manuscript.
